# In Situ Observation of Liquid Solder Alloys and Solid Substrate Reactions Using High-Voltage Transmission Electron Microscopy

**DOI:** 10.3390/ma15020510

**Published:** 2022-01-10

**Authors:** Xin F. Tan, Flora Somidin, Stuart D. McDonald, Michael J. Bermingham, Hiroshi Maeno, Syo Matsumura, Kazuhiro Nogita

**Affiliations:** 1 Nihon Superior Centre for the Manufacture of Electronic Materials (NS CMEM), School of Mechanical and Mining Engineering, The University of Queensland, St. Lucia, QLD 4072, Australia; xin.tan@uq.edu.au (X.F.T.); flora@unimap.edu.my (F.S.); s.mcdonald1@uq.edu.au (S.D.M.); 2 Advanced Materials Processing and Manufacturing (AMPAM), School of Mechanical and Mining Engineering, The University of Queensland, St. Lucia, QLD 4072, Australia; m.bermingham@uq.edu.au; 3 Centre of Excellence Geopolymer and Green Technology, Faculty of Chemical Engineering Technology, Universiti Malaysia Perlis (UniMAP), Taman Muhibbah, Jejawi, Arau 02600, Perlis, Malaysia; 4 The Ultramicroscopy Research Center, Kyushu University, Fukuoka 819-0395, Japan; maeno.hiroshi.551@m.kyushu-u.ac.jp (H.M.); matsumura.syo.819@m.kyushu-u.ac.jp (S.M.); 5 Department of Applied Quantum Physics and Nuclear Engineering, Kyushu University, Fukuoka 819-0395, Japan

**Keywords:** Pb-free solders, high voltage transmission electron microscopy, Cu_6_Sn_5_

## Abstract

The complex reaction between liquid solder alloys and solid substrates has been studied ex-situ in a few studies, utilizing creative setups to “freeze” the reactions at different stages during the reflow soldering process. However, full understanding of the dynamics of the process is difficult due to the lack of direct observation at micro- and nano-meter resolutions. In this study, high voltage transmission electron microscopy (HV-TEM) is employed to observe the morphological changes that occur in Cu_6_Sn_5_ between a Sn-3.0 wt%Ag-0.5 wt%Cu (SAC305) solder alloy and a Cu substrate in situ at temperatures above the solidus of the alloy. This enables the continuous surveillance of rapid grain boundary movements of Cu_6_Sn_5_ during soldering and increases the fundamental understanding of reaction mechanisms in solder solid/liquid interfaces.

## 1. Introduction

In compliance with the Restriction of Use of Certain Hazardous Substances (RoHS) Directive of the European Community, Pb-free solders have been developed. Today, as the primary Pb-free solder used in the electronics industry, the Sn-3.0 wt%Ag-0.5 wt%Cu (SAC305) solder has found widespread application in electronic interconnects due to its relatively low solidus and liquidus at 217 °C and 220 °C, respectively [1]. The soldering process involves heating the solder alloy above its melting temperature to react with the Cu substrate to form an intimate bond. During this process, Cu_6_Sn_5_ and Cu_3_Sn intermetallic compounds (IMCs) form at the interface between the solder melt and the Cu substrates [2,3]. The reactions at the interfaces between liquid solder/Cu_6_Sn_5_/Cu_3_Sn/solid substrates are complex. Since understanding the properties of these IMCs is critical for improving solder joint reliability, much research has been conducted using microscopy techniques at room temperature or below the solidus temperature of the solder [4,5,6]. Using in situ heating/cooling high voltage transmission electron microscopy (HV-TEM) techniques, the stabilities and phase transformation kinetics of Cu_6_Sn_5_ in solder joints have been studied by Somidin et al. [4,5,6]. However, the requirement for a solid sample makes TEM observations difficult at high temperatures where the reactions take place. While in situ synchrotron X-ray imaging has revealed the reactions between liquid solders and Cu substrates at micrometer scales [7,8], the nano- and submicron- level observations and understanding of the reactions at the liquid/solid interface remain unresolved. Creative experimental setups, including fast dipping and pulling a Cu coupon into and from a solder bath [9], quenching the joint in liquid nitrogen [10] and removing the molten solder by centrifugal forces [11], have been used to interrupt the reactions at different stages during the soldering process, providing snapshots of the high temperature microstructures. However, full understanding of the dynamics of the process is difficult due to the lack of continuous direct observation. In situ TEM observation of the solid-liquid interface has been achieved in Al-Si systems, relying on the Al oxide film to contain the liquid despite the high vapor pressures in the Al-Si system [12]. The solid-liquid interfaces of pure Al, In and Sn, along with alumina and a few binary alloys [13], Pb-Sn [14] and Pb-free solders [15] on Ni-P substrates were also studied by the same group under in situ heating TEM. TEM observations of nanometer-sized Sn–Bi [16,17] and pure metal [18] particles in the liquid state have also been achieved. In this study, qualitatively, but for the first time, using HV-TEM, we observed the morphological changes in Cu_6_Sn_5_ that has formed through a reaction of SAC305 and a Cu substrate via in situ observations at elevated temperatures up to a peak of 218 °C, just above the solidus of SAC305.

## 2. Materials and Methods

The TEM experiment was conducted with a HV-TEM (JEM-1300NEF JEOL, Akishima, Japan) at an accelerating voltage of 1250 kV. At this voltage, the beam induced heating due to inelastic scattering is less than what occurs in conventional TEMs due to the smaller interaction cross-section [4,6]. A double-tilt heating TEM holder (EM-HSTH JEOL, Akishima, Japan) equipped with a thermocouple in contact with a resistive heating element was used to heat the sample and to tilt the sample to appropriate crystal zone axes. Since the Cu_6_Sn_5_ grains have a size range between 2–5 μm, thinning the TEM lamellar to <200 nm as limited by conventional TEMs operating between 200–300 kV would result in a thin film sample where edge effects may dominate. Melting point depression also becomes increasingly significant below 100 nm [18,19]. Therefore, in this study, high voltage TEM was used to enable a sample thickness of approximately 500 nm to maintain a thickness: grain size ratio above 1:10.

A 500 μm diameter SAC305 solder ball (supplied by Nihon Superior Co. Ltd., Osaka, Japan) was reflow soldered on a Cu pad with organic solderability preservative surface finish (OSP-Cu) to fabricate a Cu/IMCs/SAC solder joint. The joint was embedded in epoxy resin and polished with standard metallographic procedures for scanning electron microscopy (SEM) of the cross-section. SEM and energy dispersive X-ray spectroscopy (EDS) elemental mapping were performed on a Hitachi TM3030 tabletop SEM at an accelerating voltage of 15 kV. Images were taken in backscattered electron mode (BSE).

**Figure 1:**
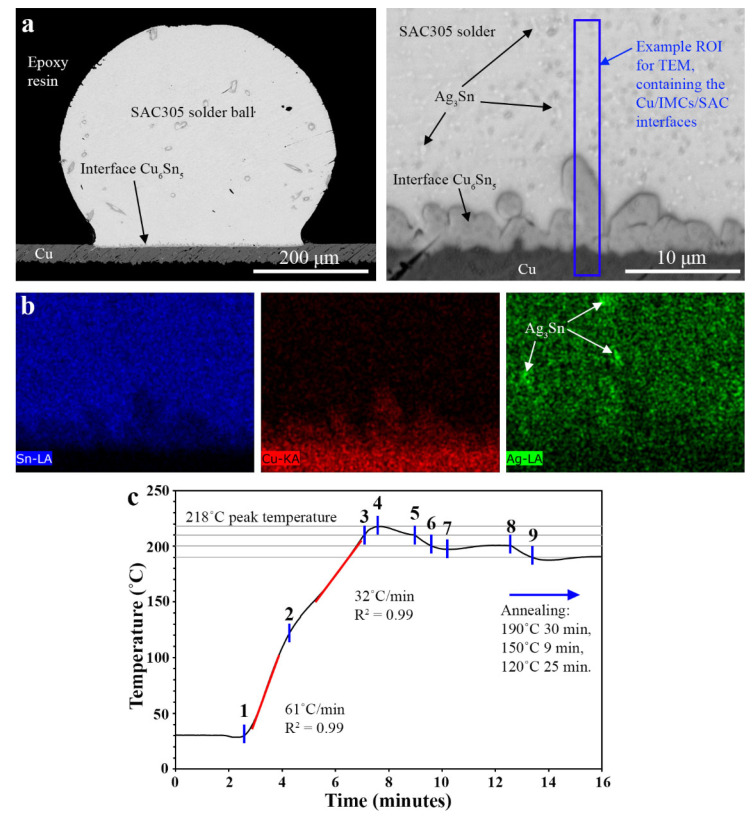
(**a**) SEM cross-section of the as reflowed Cu/IMCs/SAC solder joint at two different magnifications. (**b**) Sn, Cu and Ag EDS maps of the Cu/IMCs/SAC solder joint. (**c**) Temperature profile of the heating experiment, showing different time intervals (points 1–9) through the heating cycle.

A second Cu/IMC/SAC joint was subsequently annealed at 150 °C for 500 h to ensure a full transformation from the high temperature *η*-Cu_6_Sn_5_ crystal structure to the room temperature *η*′-Cu_6_Sn_5_ crystal structure. The sample was then cooled to room temperature in air, mounted in epoxy resin and polished with standard metallography procedures to expose the cross-sectioned microstructure. A TEM lamellar of approximately 16 μm × 25 μm was prepared using a focused ion beam (FIB) technique on a FEI Scios FIB—dual beam scanning electron microscope (SEM), similar to a technique described elsewhere [20]. The sample was extracted from a region of interest (ROI) at the Cu/IMCs/SAC interface (Figure 1a) and welded to a Cu TEM half grid by Pt deposition. Subsequently, the area of the lamellar containing the Cu/IMCs/SAC interfaces were thinned to 500 nm.

The TEM lamellar was placed on the double-tilt heating TEM holder. From the TEM viewing screen, a Cu_6_Sn_5_ grain was selected. To enhance the image contrast in the thick (500 nm) sample, an in-column omega-type filter was used to filter the plasmon contributions to the image [21,22]. The sample was tilted to a low-index zone axis of this grain and selected area electron diffraction (SAED) patterns were obtained. The sample was then heated according to the temperature profile shown in Figure 1c. A high-resolution video recorder is used to capture the evolution of the selected Cu_6_Sn_5_ grain during the heating experiment at a frame rate of 1 frame per second. The sample was held at 190 °C for 30 min, followed by 9 min at 150 °C and 25 min at 120 °C before it was cooled to room temperature. Diffraction pattern of the on-zone grain was obtained at room temperature. All SAED patterns were analyzed with Gatan Digital Micrograph version 3.20.1314.0 (Gatan, Inc., Pleasanton, CA, USA) and SingleCrystal version 2.3.3 (CrystalMaker Software Ltd., Begbroke, UK), and the corresponding crystal structures were visualized with CrystalMaker version 9.2.8.

## 3. Results and Discussion

The SEM cross-section of the SAC305 solder ball reflow soldered on to the OSP Cu pad is shown in Figure 1a along with a higher magnification image displaying the Cu/IMCs/SAC interface. The Cu_6_Sn_5_ IMC has a scalloped-like morphology. Under BSE mode, the Sn-rich SAC305 solder matrix has a good contrast with the Cu_6_Sn_5_ IMC and the Cu substrate due to the differences in the averaged atomic numbers of approximately 50, 38.5 and 29, respectively. Ag has an atomic number of 47 which is relatively close to the atomic number of Sn, therefore the contrast between Ag_3_Sn and the Sn-rich matrix is low. The brighter spots in the Ag-LA EDS map in Figure 1b corresponds to the fine Ag_3_Sn plates, while the Sn-LA and Cu-KA maps help identify the Sn-rich SAC305 solder, the Cu_6_Sn_5_ IMC and the Cu substrate. The Cu_3_Sn IMC layer in the as reflowed joint was thin and was not visible due to the resolution limit of the SEM. The observed microstructure agrees with those widely reported in soldering literature [23,24].

**Figure 2:**
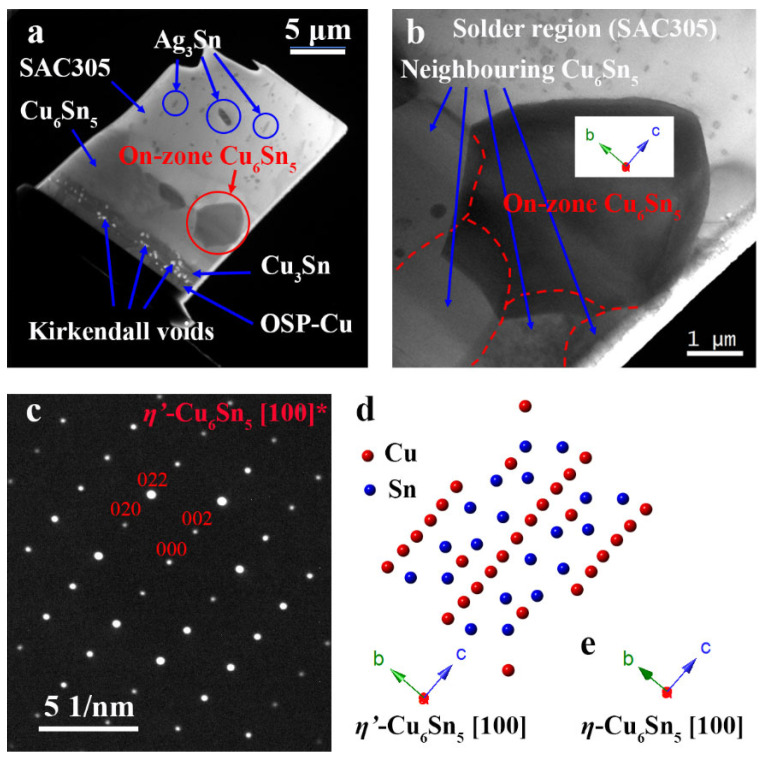
(**a**) Low magnification on-zone bright-field (BF) plasmon filtered TEM image of the lamellar before the heating experiment. (**b**) Image of the on-zone Cu_6_Sn_5_ on top of four other Cu_6_Sn_5_ grains, and (**c**) the SAED pattern of the on-zone grain (**d**) a schematic of the crystal structure showing the crystal orientation, and (**e**) the equivalent orientation in a *η*-Cu_6_Sn_5_ crystal to produce the diffraction pattern indexed in (**c**).

Figure 2a,b show the TEM lamellar and an on-zone Cu_6_Sn_5_ grain, respectively. The Cu_3_Sn layer thickened as a result of ageing at 150 °C for 500 h [2,3,23]. Kirkendall voids, which often accompany the formation of Cu_3_Sn [3], are present between the Cu_3_Sn and the OSP-Cu (Figure 2a). The selected grain is not directly connected to the OSP-Cu/Cu_3_Sn, and the boundaries between the on-zone grain and four other Cu_6_Sn_5_ grains are outlined in Figure 2b. The selected area electron diffraction (SAED) pattern of this grain in Figure 2c is indexed to the [100] zone axis of *η*′-Cu_6_Sn_5_ (monoclinic, C2/c, International Centre for Diffraction Data, ICDD number: 047-1575) crystal structure, at the crystal orientation shown in Figure 2d. The Cu_6_Sn_5_ grain grew with the c-axis of the equivalent *η*-Cu_6_Sn_5_ crystal structure (hexagonal, P6_3_/mmc, International Centre for Diffraction Data, ICDD number: 045-1488) perpendicular to the surface of the OSP-Cu, which is consistent with the preferred orientation reported in the literature [11].

**Figure 3:**
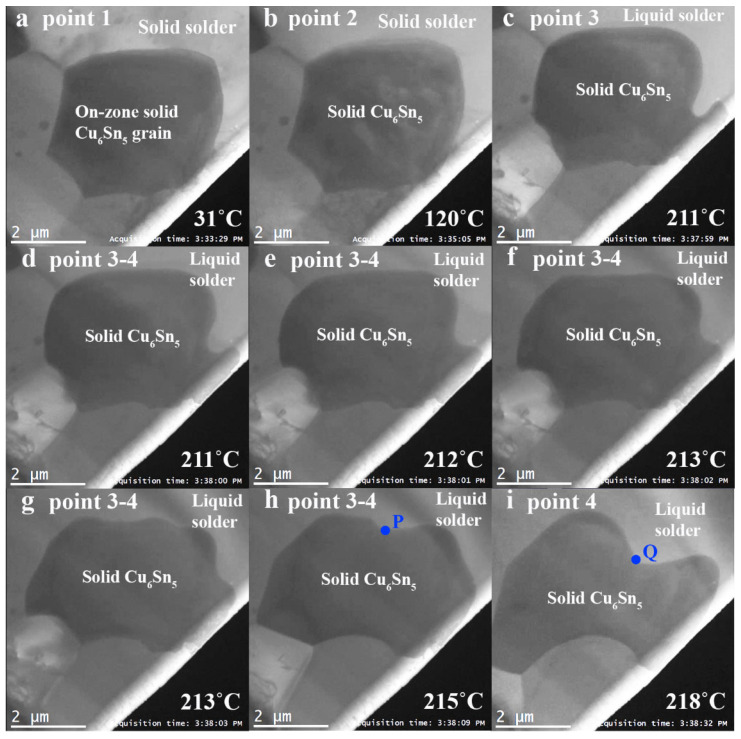
Snapshots of the heating experiment at each of the time intervals indicated in Figure 1c: (**a**) point 1, (**b**) point 2, (**c**–**g**) 1-s interval snapshots over a 4 s period highlighting the rapid grain boundary movements starting from point 3, (**h**) between point 3 and 4 at 215 °C and (**i**) point 4.

Video S1 (Supplementary Materials) shows the recorded video during the heating experiment that is accelerated 20 times. Between point 1 and 2, the sample was heated to 120 °C at a rate of 61 °C/min. Between point 2 and 3, the heating rate was reduced to 32 °C/min to reduce temperature overshoot. It is expected that the Cu_6_Sn_5_ grains transform from a monoclinic *η*′ crystal structure to a *η* crystal structure around 186 °C [25] during this stage as observed in published TEM works [4,6]. During these heating stages, the selected Cu_6_Sn_5_ grain grew gradually (Figure 3a–c) until the temperature reached 211 °C at point 3, where rapid changes in the morphology of the solid Cu_6_Sn_5_ were observed (Figure 3c–i). Figure 3c–g are 1-s interval snapshots over a 4 s period highlighting the rapid grain boundary movements starting from point 3. It is believed that at this point the SAC305 solder (solidus at 217 °C) has started to melt while the Cu_6_Sn_5_ grain remained solid (solidus at 408 °C). The presence of liquid solder at this temperature is expected to enhance diffusion rates within the solder. The solidus is lower than the expected 217 °C, potentially due to electron beam heating. Since the metallic sample and the sample stage have good thermal conductivities, it is expected that the difference between the real sample temperature and the read-out temperature is small, though this may have also contributed to the sample melting at a lower read-out temperature. The sample appears to be self-sustaining despite the presence of liquid likely due to the mechanical strength imparted by the surface tension of the thin oxide film (581 mN m^−1^ at the melting point of Sn [26]), similar to the case in the Al-Si experiment conducted by Howe and Saka [12]. Furthermore, Sn has a vapor pressure at the order of 1 × 10^−20^ Pa close to its melting point [27], that is orders of magnitude lower than the vapor pressure of Al alloys at the order of 7 × 10^−9^ Pa at the Al-Si eutectic of 577 °C [28] and is less likely to vaporize under the TEM vacuum. The dark contrast of the selected Cu_6_Sn_5_ grain is evidence that the grain remained solid throughout the heating experiment, as it continues to diffract the electron beam. The fast movement at the Cu_6_Sn_5_/SAC interface, measured up to 2.5 μm/s (movement of point P to point Q in Figure 3h,i), is due to the dissolution of the solid Cu_6_Sn_5_, as observed during a second reflow in an ex-situ experiment performed by Gong et al. [11]. On the other hand, at the bottom left of the grain, which is closer to the Cu_3_Sn layer, grain growth towards the direction of the Cu source was observed.

**Figure 4:**
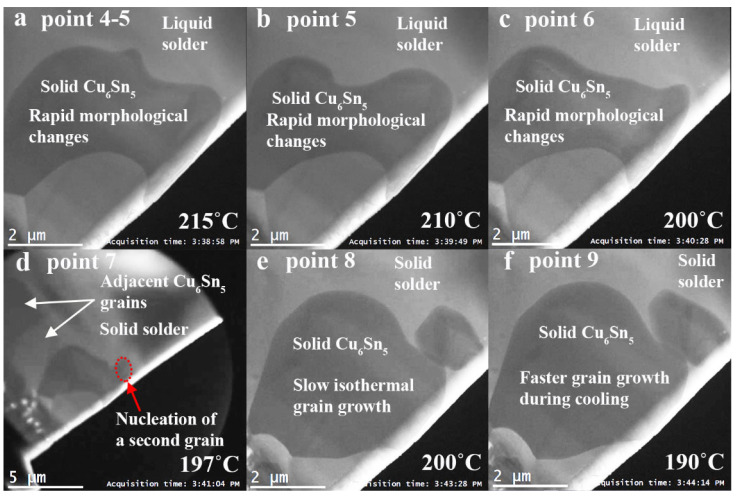
Snapshots of the heating experiment (**a**) between point 4 and 5, (**b**) at point 5, (**c**) point 6, (**d**) point 7 (zoomed out to provide a view of the area surrounding the selected grain), (**e**) point 8 and (**f**) point 9.

Sample heating was stopped shortly after point 3 after the rapid morphological changes were observed. The temperature overshot to 218 °C at point 4, before cooling to a set temperature of 210 °C at point 5 and then 200 °C at point 6, and was maintained at this near-isothermal temperature for 180 s. The rapid grain boundary movements continued through from point 3 to point 7 (Figure 3c–i and Figure 4a–c). A second Cu_6_Sn_5_ grain nucleated at point 7 (Figure 4d), where a minimum temperature at 197 °C was measured. In Figure 4d, the video is zoomed out to a lower magnification to provide a view of the area surrounding the selected Cu_6_Sn_5_ grain, where the grain boundaries of two adjacent grains are visible to the top-left of the selected grain. The morphological changes became sluggish from this point onwards (Figure 4d,e), as the SAC305 solder solidified. At this stage, the bottom left of the Cu_6_Sn_5_ grain has grown to less than 1 μm from the Cu_3_Sn. From point 8 to 9, the sample was cooled to 190 °C. During cooling, the growth rate of the two Cu_6_Sn_5_ grains accelerated (Figure 4e,f), as the dissolution limit of Cu in the SAC alloy reduced with temperature [11], and the excess Cu was deposited onto the Cu_6_Sn_5_ grains. Gong et al. [11] state that the heterogeneous dissolution and growth may have resulted in the scallop morphology of the Cu_6_Sn_5_ phase. The in situ observation in this study shows the Cu_6_Sn_5_ grain does not have a scallop morphology during dissolution, and the scallop morphology is formed when Cu is deposited onto the Cu_6_Sn_5_ grains during cooling. 

The Gibbs–Thomson effect results in chemical potential variations across the curved interface between the solid Cu_6_Sn_5_ and liquid solder which can lead to undercooling [29,30]. The concentration of Cu in liquid solder at the localized surface of the grain, *C_r_*, is given by [31]

(1) $$C_{r}≅\;C_{0}\;\left(1+\frac{2\gamma_{SL}\Omega }{rRT}\right),\;\mathrm{when}\;\frac{2\gamma_{SL}\Omega }{rRT}\ll 1,$$

where *C*_0_ is the concentration of Cu in the solder, *γ_SL_* is the solid-liquid interfacial energy, Ω is the molar volume of Cu_6_Sn_5_, *R* is the gas constant, *T* is the temperature, and *r* is the radius of curvature which is positive for a convex grain interface and negative for a concave grain interface [32]. Therefore, a convex Cu_6_Sn_5_ interface of small curvature radius *r*, will increase the local concentration of Cu while a concave surface will result in a local Cu concentration that is lower than the Cu concentration in the solder. As a result, apart from the Cu fluxes from Cu_3_Sn and the adjacent Cu_6_Sn_5_ grains which promotes grain growth and Ostwald ripening [31,33], there is also Cu flux exchange at different local interfaces within a grain due to the difference in curvature radii, which may have contributed to the rapid changes in morphology at the Cu_6_Sn_5_/liquid interface. The Cu flux, *J*, can be calculated by the following equation [31],

(2) $$J=-D\frac{C_{r1}-C_{r2}}{L}=-\frac{2\gamma_{SL}\Omega DC_{0}}{LRT}\left(\frac{1}{r_{1}}-\frac{1}{r_{2}}\right),$$

where *D* is the diffusivity of Cu in liquid solder, *C_r_*_1_ and *C_r_*_2_ are the Cu concentrations in liquid solder at two localized point surfaces calculated by Equation (1), *L* is the distance between the two points, and *r*_1_ and *r*_2_ are the radii of curvature at point 1 and point 2 respectively.

**Figure 5:**
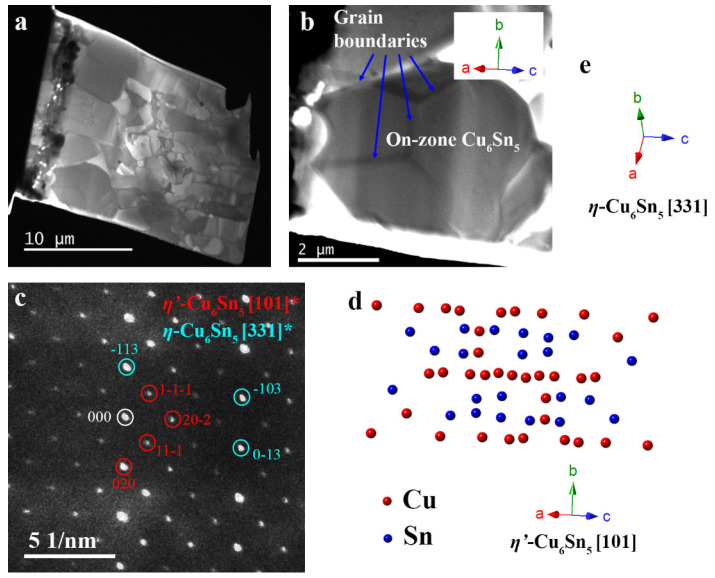
(**a**) Low magnification image of the lamellar after the heating experiment, (**b**) image of the on-zone Cu_6_Sn_5_ grain after tilting, (**c**) the SAED pattern and (**d**) a schematic of the crystal structure of the on-zone *η*′-Cu_6_Sn_5_ grain showing the grain orientation, and (**e**) the equivalent orientation in a *η*-Cu_6_Sn_5_ crystal to produce the diffraction pattern indexed in (**c**).

After the real-time observation, the sample was held at 190 °C for 30 min. Again, slow grain growth was observed during this period. This was followed by 9 min at 150 °C and 25 min at 120 °C before the sample was cooled to room temperature (Figure 5a,b). The sample was again tilted to a low index zone axis of the selected Cu_6_Sn_5_ grain and the SAED pattern was captured and indexed (Figure 5c). As indicated by the monoclinic reflections in Figure 5c, the grain has the crystal structure of a *η*′-Cu_6_Sn_5_, which is expected as the annealing stages allowed the high temperature *η* to fully convert into *η*′ as observed by Somidin et al. under HV-TEM [4,5]. The grain orientation is unchanged (Figure 5b,c), with the c-axis of an equivalent *η*-Cu_6_Sn_5_ crystal perpendicular to the OSP-Cu (Figure 5c,e). The low resolution plasmon filtered image of the sample in Figure 5a shows significant growth in all Cu_6_Sn_5_ grains in the direction perpendicular to the OSP-Cu. In contrast, no Cu_6_Sn_5_ grain coalescence was observed and there is no lateral growth.

After 30 min of annealing at 190 °C, part of the selected Cu_6_Sn_5_ grain has connected to the Cu_3_Sn phase. The growth in this direction continued during subsequent annealing at 150 °C and 120 °C, resulting in the final microstructure shown in Figure 5a,b. Again, the Ostwald ripening growth model [31,33] was not observed and there is no lateral growth during the annealing stages. Lord et al. [9] reported that lateral growth begins after the concentration of Cu in the solder reaches the dissolution limit. It is likely that the SAC305 in this experiment was not saturated due to the relatively short time above the melting temperature of the solder, which explains the lack of lateral growth.

## 4. Conclusions

The presented results are the first direct in situ observation of the dissolution of Cu_6_Sn_5_ at the solid Cu_6_Sn_5_/liquid solder interface and Cu_6_Sn_5_ growth at the Cu_6_Sn_5_/Cu_3_Sn interface at these temperatures. With this, the singular behavior of the solid/liquid interface with a weak surface tension is disclosed. That is, the phase boundary between Cu_6_Sn_5_ and SAC305 softened significantly when the latter is in a molten state, likely owing to repeated partial dissolution and re-solidification of Cu_6_Sn_5_ into and from SAC305.

The observation is consistent with published snapshots of the process based on ex-situ techniques in Gong et al.’s work, using a Sn-Ag-Cu alloy with slightly higher alloying contents i.e., Sn-3.8 wt%Ag-0.7 wt%Cu [11]. As long as the composition of the solder is below the solubility limits, Cu_6_Sn_5_ dissolves into the solder resulting in rapid changes in grain morphology above the solidus of the solder. Furthermore, the growth of Cu_6_Sn_5_ in the direction of Cu_3_Sn (Cu source) is substantial during soldering at high temperature; while the growth in the direction of the solder happens during cooling due to the deposition of Cu from the melt as the solubility of Cu reduces with decreasing temperature. This observation can help in designing strategies to reduce the thickness of the brittle Cu_6_Sn_5_ IMCs which are a primary failure point in solder joints and improve joint reliability, for example, by applying a temperature gradient to drive the diffusion of Cu away from the IMC/liquid interface and by replenishing the solder bath during wave soldering with solder alloys of lower Cu to maintain the Cu concentration in the melt.

This method for observing solid/liquid interaction using HV-TEM is likely to find application for other alloy/substrate systems such as those found in coating or welding processes to provide further understandings of the mechanisms involved. While the HV-TEM method allows for the use of thicker samples and edge-effects are less likely to influence the observed microstructural changes, further work is required to determine how closely these processes approach those occurring in bulk samples of industrially significant length scales.

## Data Availability

The data presented in this study are available on request from the corresponding author.

## Supplementary Materials

The following are available online at www.mdpi.com/article/10.3390/ma15020510/s1, Video S1: The recorded video accelerated 20 times.
